# Identifying Active Compounds and Mechanism of *Camellia nitidissima* Chi on Anti-Colon Cancer by Network Pharmacology and Experimental Validation

**DOI:** 10.1155/2021/7169211

**Published:** 2021-08-26

**Authors:** Yiwei Chen, Erwei Hao, Fan Zhang, Zhengcai Du, Jinling Xie, Feng Chen, Chunlin Yu, Xiaotao Hou, Jiagang Deng

**Affiliations:** ^1^School of Pharmacy, Chengdu University of Traditional Chinese Medicine, Chengdu 611137, Sichuan, China; ^2^Guangxi Key Laboratory of Efficacy Study on Chinese Materia Medica, Guangxi University of Chinese Medicine, Nanning 530200, Guangxi, China; ^3^Collaborative Innovation Center for Research on Functional Ingredients of Agricultural Residues, Guangxi University of Chinese Medicine, Nanning, Guangxi 530200, China; ^4^Postdoctoral Workstation, Guangxi Institute of Medicinal Plants, Nanning 530023, China

## Abstract

Camellia *nitidissima* Chi (CNC) is a traditional Chinese medicine (TCM) with anticancer property. However, its underlying mechanisms of anti-colon cancer (CC) remain unknown. Therefore, a systematic approach is proposed in the present study to elucidate the anticancer mechanisms of CNC based on network pharmacology and experimental validation. Initially, the potential active ingredients of CNC were verified via the TCMSP database based on the oral bioavailability (OB) and drug-likeness (DL) terms. Hub targets of CNC were acquired from SwissTarget prediction and TCMSP databases, and target genes related to CC were gathered from GeneCards and OMIM databases. Cytoscape was used to establish the compound-target networks. Next, the hub target genes collected from the CNC and CC were parsed via GO and KEGG analysis. Results of GO and KEGG analysis reveal that quercetin and luteolin in CNC, VEGFA and AKT1 targets, and PI3K-Akt pathway were associated with the suppression of CC. Besides, the result of molecular docking unveils that VEGFA demonstrates the most powerful binding affinity among the binding outcomes. This finding was successfully validated using *in vitro* HCT116 cell model experiment. In conclusion, this study proved the usefulness of integrating network pharmacology with *in vitro* experiments in the elucidation of underlying molecular mechanisms of TCM.

## 1. Introduction

Colon cancer (CC) is a type of malignant tumor with high incidence and mortality worldwide [1]. It is characterized high recurrence, fast growth and migration, and poor prognosis [2–4]. Surgery, chemotherapy, and radiotherapy treatment are the three primary types of treatment for CC. Although these treatments are typically effective, they often cause side effects such as toxicity and gastrointestinal irritation. Therefore, it is exceptionally urgent to develop safe and novel medicines for the treatment of CC.

Traditional Chinese medicine (TCM) has gained popularity around the world due to its fewer side effects and reliable efficacy [5]. With the present advanced research and clinical trial in the field of biochemistry and pharmacology, TCM is recognized as an excellent source of complementary and alternative anticancer drugs. *Camellia nitidissima* Chi (CNC) is a wild woody plant that is widely used as a traditional folk medicine to effectively treat various diseases, such as hypertension, hyperlipidemia, and infection [6]. Moreover, numerous modern pharmacological researches proved that CNC has significant inhibitory effects on various cancers [7–10]. Even though the evidence to support the medicinal claim of CNC is widely reported, its anticancer mechanism remains unclear due to its multitarget and multicomponent characteristics.

With the rapid progress of bioinformatics, network pharmacology which was first proposed by Cao et al. in 2007 is proven as a powerful tool in the exploratory research of TCM [11–13]. The strength of network pharmacology in delivering a systematic understanding of drug action and disease complexity effectively develops the pharmacological model which could comprehensively demonstrate the effects of TCM on particular diseases [14–17]. Therefore, the present study integrated network pharmacology with *in vitro* experimental validation to elucidate the mechanism of action of CNC against CC. At the same time, it is the first time systematically studying the anti-CC effect of CNC by using network pharmacology combined with an *in vitro* experimental model. It provides a scientific basis for elucidating the mechanism of CNC in the treatment of CC and provides a new idea for more in-depth research.

**Figure 1:**
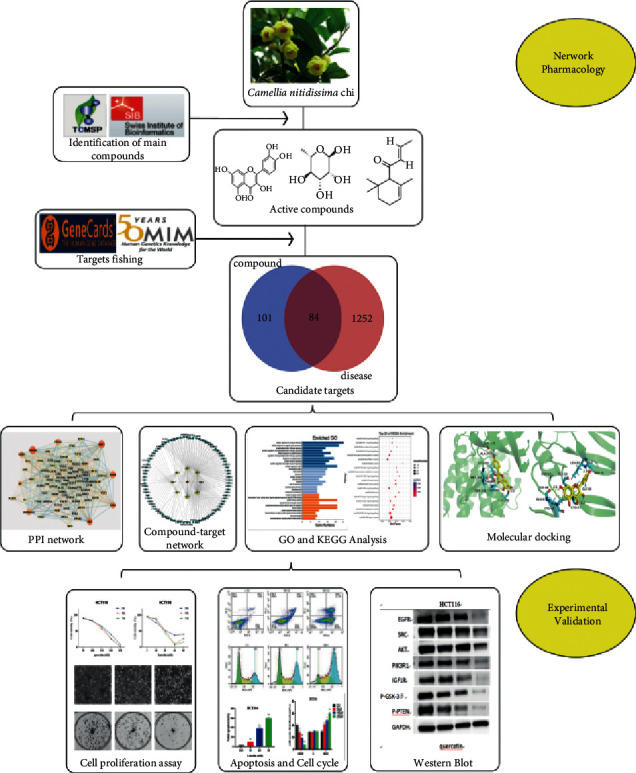
The detailed flow chart of the current study.

Briefly, targets for active compounds of CNC and CC were collected through TCMSP and SwissTarget prediction platforms, whereas the intersection targets were identified by constructing a Venn diagram. Next, a protein-protein interaction (PPI) network diagram of intersection proteins was constructed, followed by Gene Ontology (GO) and Kyoto Encyclopedia of Genes and Genomes (KEGG) enrichment analysis. Lastly, a “compound-target-pathway” network diagram was constructed by using Cytoscape software and molecular docking was performed using AutoDock. Experimental evidence shows that quercetin and luteolin in CNC, VEGFA and AKT1 targets, and PI3K-Akt pathway were involved in the suppression of CC by blocking the HCT116 cycle and inducing apoptosis. This research provides evidence to support the clinical use of CNC in CC treatment. The detailed flow chart of the current study is as follows (Figure 1).

## 2. Materials and Methods

### 2.1. Network Pharmacology

#### 2.1.1. Screening of the Active Ingredients of CNC

The main chemical components of CNC were identified via literature search using CNKI and PubMed databases. The corresponding chemical structure, chemical English name, CAS number, and other information of the compounds were obtained from the chemical source network (https://www.chemsrc.com/) and PubChem [18] (https://pubchem.ncbi.nlm.nih.gov/). In the TCM systems pharmacology Database and Analysis Platform (TCMSP), the chemical constituents of CNC were searched. According to the standard of TCMSP [19], the values of oral bioavailability (OB) and drug-likeness (DL) were used as the indexes for screening the compounds of CNC, and those compounds with DL ≥0.18 and OB ≥ 30% were selected as the active constituent of CNC. For the compounds that were not found in the TCMSP database, their 2D chemical structural formulas were searched and downloaded from PubChem. The SDF format of the compounds was imported into Swiss Target prediction database [20] (http://www.swisstargetprediction.ch/) for screening of the active components (https://www.uniprot.org/) in CNC. UniProt database [21] (https://www.uniprot.org/) was used to normalize the target names in the predicted target library.

#### 2.1.2. Identification of Potential Targets of Active Ingredients

The 2D chemical molecular structure formula (in .SDF format) of active chemical compounds was retrieved and exported from the PubChem to SwissTarget prediction database with the species selection of “*Homo sapiens*,” and the predicted results were exported in .CSV format. Active ingredients whose targets were not successfully predicted through the SwissTarget prediction database were searched in the TCMSP database, and the predicted targets were derived. By combining results obtained from these two databases, the predicted target library of active ingredients of CNC was obtained.

#### 2.1.3. Identification of Anti-CC Targets

The search term “colon cancer” was input into the human genes and genetic phenotypes comprehensive database [22] (OMIM, https://www.omim.org/) and the annotation of the human genome (GeneCards, https://www.genecards.org/) database to identify CC-related genes. GeneCards database is a platform that can provide the proteome, transcription, inheritance, and function of all known genes in the human genome. By combining the genes obtained from the two databases after removing the duplicates, protein targets associated with CC were identified. With the help of the Draw Venn Diagram (http://bioinformatics.psb.ugent.be/webtools/Venn/) website and the targets obtained in Section 2.1.2, the common target genes obtained were identified as the potential targets of CNC to suppress CC.

#### 2.1.4. Herbal-Compound-Target Network Construction

The potential targets obtained in Section 2.1.3 were imported into the Cytoscape 3.7.2 software [23] to construct the herbal-compound-target network to visualize the relationship between the compound and the target protein. The “nodes” in the network represent the corresponding compounds and target proteins, the “edges” define the relationship between compounds and target proteins, and the degree value represents the number of edges connected to a node. The larger the value, the more critical the nodes are in the network, and the “Network Analyze” function was used to analyze its topology properties.

#### 2.1.5. PPI Network Analysis

The potential targets of CNC against CC identified in Section 2.1.3 were imported into the String database (https://string-db.org/) [24], and the species was limited to “*Homo sapiens*,” while other parameters were set at a default value. Eventually, a target interaction network diagram was obtained and saved in .tsv format. The above data were then imported into Cytoscape3.7.2 software for visual processing. Lastly, the “Network Analyzer” tool was used to analyze the network to obtain the degree value (connectivity) and draw the PPI network diagram. The network was exported in PNG format and the top 10 hub genes were selected.

#### 2.1.6. GO and KEGG Pathway Analysis

Metascape [25] (https://metascape.org/) is a bioinformatics database that integrates biological data and analytical tools for GO enrichment analysis and KEGG pathway analysis. The potential targets identified in Section 2.1.3 were imported into the Metascape database and screened with *p* < 0.01 as the critical value of significant functions and pathways. The main signal pathways and biological processes involved in the pharmacological effects of CNC against CC were obtained. The Cytoscape3.7.2 software was used to construct the “compound-target-pathway” network diagram of the signal pathway obtained in the pathway enrichment analysis to realize the visualization research and analyze the multicomponent, multitarget, and multipathway action mechanisms of CNC against CC.

#### 2.1.7. Molecular Docking

Molecular docking is an important method of computer-aided drug design. Intermolecular interaction recognition is carried out by simulating the geometric structure and intermolecular force of molecules to predict the structure of receptor-ligand complexes. The crystal structures of the candidate targets were downloaded from RCSB Protein Data Bank (http://www.pdb.org/) [26] and embellished through the PyMOL 2.3.4 software by removing the ligands, adding hydrogen, and removing water. The three-dimensional chemical structural formulas of ligands were obtained from the PubChem database and converted into PDB file using PyMOL 2.3.4 software. Docking was carried out by using AutoDock software, whereas PyMOL and LigPlot 2.2 software were used to visualize the optimal configuration.

### 2.2. Experimental Validation

#### 2.2.1. Sample Preparation

A total of 25 g dried CNC (from Fangchenggang, Guangxi) was weighed and then immersed in 0.625 l of 75% (v/v) ethanol for 30 min. Reflux extraction was carried out for 1.5 h twice. The extracts from two stages of extraction were combined and concentrated by using a rotary evaporator. Next, the concentrated extract was freeze-dried to obtain powder (3.66 g). DMSO was used to dissolve the powder in 100 mg/kg concentration and filtered by a 0.22 *μ*m filter membrane and then frozen at −20°C. Quercetin, luteolin, kaempferide, and kaempferol with a purity of 99% were purchased from MCE.

#### 2.2.2. Cell Culture

Human CC HCT116 was selected in this study. HCT116 cell line was purchased from Procell Life Science and Technology Co., Ltd. (Wuhan, China). The cells were cultured and maintained on the McCoy's 5 A medium supplemented with 10% FBS, 100 U/ml penicillin, and 100 mg/ml streptomycin in a humidified chamber with 5% CO_2_ at 37°C.

#### 2.2.3. Cell Viability Assay and Cell Morphology

For the cell viability assay, 3,000 HCT116 cells/well were seeded in 96-well plates. After overnight incubation, the cells were treated with different concentrations of quercetin (50, 100, 150, 200, 250 *μ*M), luteolin (5, 10, 20, 40, 80 *μ*M), kaempferide (12.5, 25, 50, 100, 200 *μ*M), and kaempferol (12.5, 25, 50, 100, 200 *μ*M) for 24, 48, and 72 h, respectively. Next, 20 *μ*l of MTS (20 ml; Promega, USA) was added to each well, and the cells were cultured for another 2 h at 37°C. The absorbance was measured at 490 nm using the high-throughput microplate reader screening system (PerkinElmer, USA). The cell morphology was observed and captured by using an inverted microscope (OLYMPUS, Japan).

#### 2.2.4. Colony Formation Assay

To evaluate the colony forming ability of cells, 1,000 cells/well were seeded in 6-well plates. After overnight incubation, the cells were treated with or without quercetin (6.25, 12.5, 25 *μ*m) and luteolin (2.5, 5, 10 *μ*m) for seven consecutive days. On the fourth day, the medium was replaced with the fresh medium containing quercetin or luteolin. After fixation with 4% paraformaldehyde for 20 min, the cells were stained with crystal violet solution for 10 min. Then, the plates were rinsed with PBS, and the colonies were counted and their picture was captured by using a GelCount equipped with count analysis system.

#### 2.2.5. Flow Cytometry Analysis

For apoptosis analysis, 1.5 × 10^5^ cells/well were seeded in 6-well plates. After overnight incubation, the cells were treated with or without quercetin (50, 100, 200 *μ*M) and luteolin (10, 20, 40 *μ*M) for 48 h. The cells were collected and suspended in a 100 *μ*l binding buffer containing 5 *μ*l FITC-conjugated Annexin V and 5 *μ*l PI (Annexin V FITC Apop Dtec, BD). After dark incubation for 15 min at room temperature, a 400 *μ*l binding buffer was added. The apoptosis condition of the cell samples was analyzed using the Attune Flow cytometer (ThermoFisher, USA) within 1 h.

For cell cycle analysis, the cells were collected and fixed with 75% ethanol at −20°C overnight. Then, the cells were incubated with 0.5 ml propidium iodide (PI, 0.5 ml FxCycleTM PI/RNAse Solution, BD) in the dark for 45 min. The cell samples were tested using the Attune Flow cytometer (ThermoFisher, USA) for cell cycle analysis.

#### 2.2.6. Western Blot (WB) Analysis

For WB analysis, 1 × 10^6^ cells/well were seeded in 6-well plates. After overnight incubation, the cells were treated with or without quercetin (50, 100, 200 *μ*M), luteolin (10, 20, 40 *μ*M), and CNC extract (25, 50, 100, 150 *μ*g/ml) for 48 h. Next, the cells were collected and rinsed twice with ice-cold PBS, lysed, incubated in RIPA buffer containing a 1% protease inhibitor cocktail (ThermoFisher, USA) for 30 min on ice, and finally centrifuged at 12,000 ×g for 15 min. The supernatant was harvested, and the protein concentration was determined using a Pierce™BCA Protein Assay Kit (ThermoFisher, USA). The protein samples were then separated by 12% SDS-PAGE (Bio-Rad, USA) and then transferred to a PVDF membrane (Millipore, USA). After blocking for 1 h, the antibodies were incubated at 4°C overnight. The primary antibodies detected include EGFR, SRC, AKT, PIK3R1, and IGF1R (CST, USA). The protein bands were visualized using ECL detection reagents (Bio-Rad, USA).

#### 2.2.7. Statistical Analysis

To compare the means between the groups, a one-way ANOVA followed by the LSD-t multiple comparison test was performed. Each experiment was carried out in triplicate. The GraphPad 8.0.2 software was used to perform a two-tailed *t*-test, and *p* < 0.05 was considered statistically significant. All results were presented as mean ± standard deviation (SD).

## 3. Results

### 3.1. Network Pharmacology

#### 3.1.1. Identification of Potential Bioactive Compounds in CNC

A total of 83 compounds of CNC were retrieved from SwissTarget prediction and TCMSP databases. Among these compounds, there were eight compounds that met the OB threshold at ≥30%, and DL index at ≥0.18 (Table 1). These compounds are mainly flavonoids, at which quercetin and luteolin are among the compounds that have been long reported to exhibit prominent antitumor and anti-inflammatory activities [27, 28].

#### 3.1.2. Identification of Candidate Targets of CNC for CC Treatment

**Figure 2:**
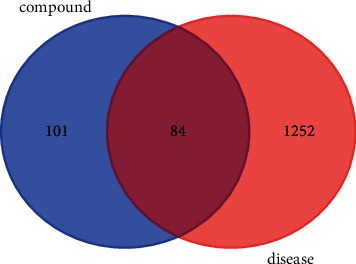
Venn diagram of drug targets and disease proteins.

Through the SwissTarget prediction database and TCMSP analysis platform, the corresponding targets of the eight active compounds of CNC were obtained. A total of 185 targets were identified after the duplicates were removed. Besides, a total of 156 CC-related targets were selected from the OMIN database and 1,271 targets were selected from the GeneCards database. After removed the duplicate targets, a total of 1,336 CC-related targets were identified. The targets were correlated, and a total of 84 targets were indicated as the potential targets.  Figure 2 shows the Venn diagram that illustrates the selection the targets.

#### 3.1.3. Herbal-Compound-Target Network Construction and PPI Analysis

**Figure 3:**
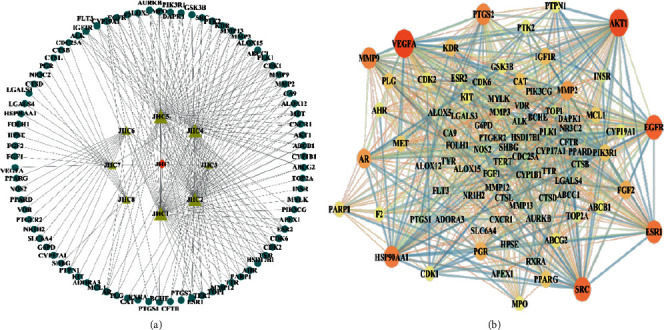
(a) Herbal-compound-target network construction. Orange quadrilaterals represent herbal; yellow triangles represent compounds; blue circles represent targets. (b) PPI network analysis.

Figure 3(a) illustrates the herbal-compound-target network of the 84 potential targets. The top four compounds with the highest degree values were quercetin (JHC1), luteolin (JHC4), kaempferide (JHC5), and kaempferol (JHC2).  Figure 3(b) demonstrates the PPI relationship (84 nodes and 702 edges). The darker the color, the more significant the interaction. According to the PPI network, the ten hub targets with the highest degree values were VEGFA, AKT1, EGFR, SRC, ESR1, HSP90AA1, MMP9, PTGS2, AR, and MMP2.

#### 3.1.4. GO Enrichment and KEGG Pathway Analysis

**Figure 4:**
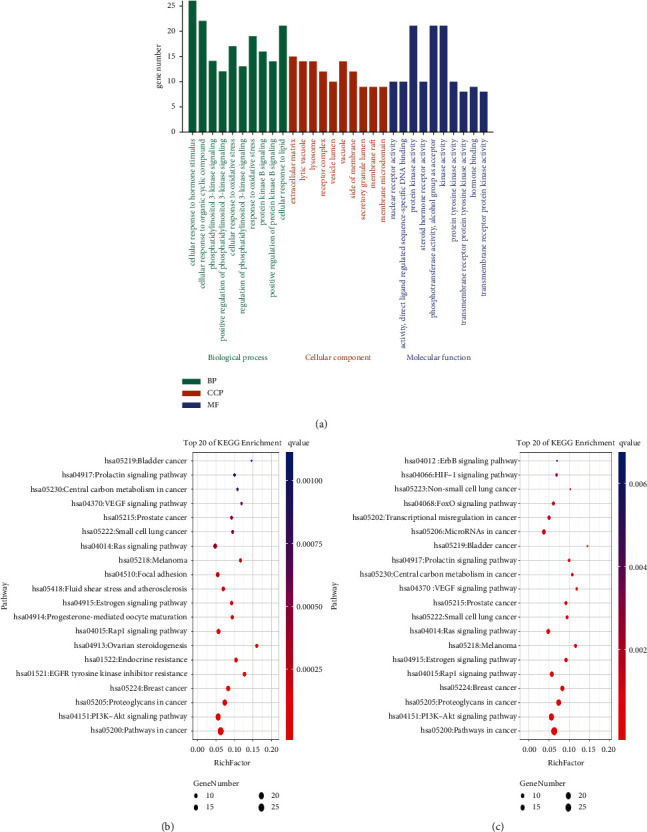
(a) The top 10 of GO enrichment analysis. (b) The top 20 of KEGG pathway enrichment analysis. (c) The top 20 of KEGG pathways related to CC.

Results of GO enrichment analysis show that the biological processes (BP) were mainly related to the cellular response to hormone stimulus, phosphatidylinositol 3-kinase signal transduction, and oxidative stress. The cell composition (CCP) is mainly included in the extracellular matrix, lysosome, and cysts. The molecular functions (MF) were mainly the nuclear receptor activity, transcription factor activity, and protein kinase activity.  Figure 4(a) shows the top ten hub targets selected after being sorted by log P. The details of the above GO entries were provided in the supplementary materials (Table S1). Based on the results of KEGG enrichment analysis, a total of 100 pathways (with *p* value <0.05) that were mainly enriched in the key targets of CNC for the treatment of CC were identified. The main pathways that were identified are cancer pathway, PI3K-Akt signaling pathway, EGFR tyrosinase inhibitor resistance pathway, Rap1 signaling pathway, etc. The top 20 pathways were then selected according to the logq value to draw a bubble chart (Figure 4(b)). In addition, the top 20 pathways related to CC (with *p* < 0.05) were also selected and presented in a bubble diagram, as shown in Figure 4(c).

#### 3.1.5. Compound-Target-Pathway Network Construction

**Figure 5:**
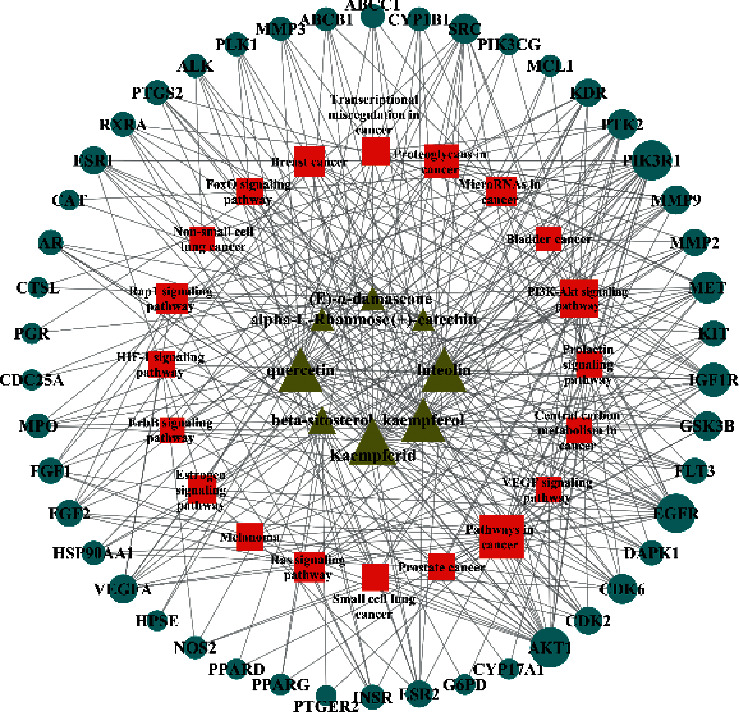
Compound-target-pathway of CNC against CC (yellow triangles represent compounds; red squares represent pathways; blue circles represent targets).

To demonstrate a better understanding on the molecular mechanism of CNC against CC, the targets of CNC and CC and the related pathways were taken as the nodes to construct the “compound-target-pathway” network diagram, as shown in Figure 5. PIK3R1, AKT1, EGFR, and IGF1R were identified as the main anticancer targets of CNC. Kaempferide, luteolin, quercetin, and kaempferol were the main active compounds that exhibit the anticancer effect in CNC.

#### 3.1.6. Molecular Docking

**Figure 6:**
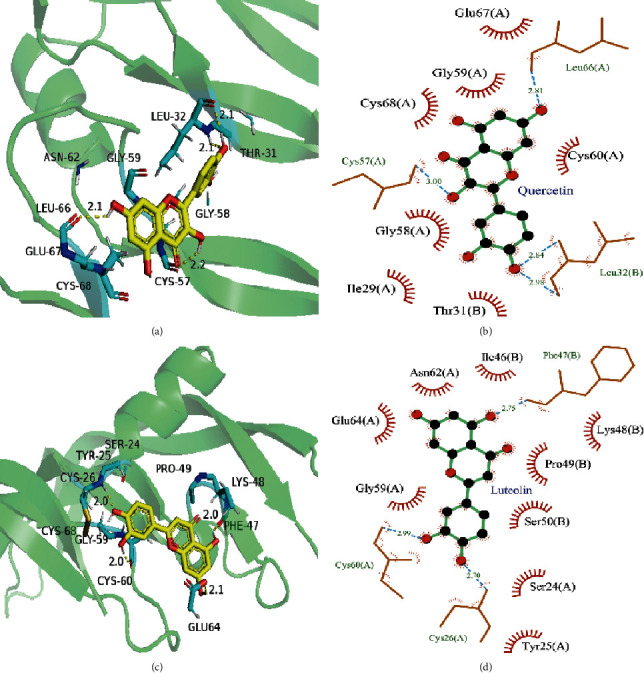
(a) Molecular model of the most active molecule in the quercetin in the protein VEGFA (PDB ID: 1MKK). Active site amino acid residues are represented as a blue stick model, while the ligand is shown as a stick model, yellow-colored. (b) Schematic (2D) representation of interactions of quercetin in the binding pocket of the protein. (c) Molecular model of the most active molecule in the luteolin in the protein VEGFA (PDB ID: 1MKK). (d) Schematic (2D) representation of interactions of luteolin in the binding pocket of the protein.

Docking studies were performed using AutoDock in the active sites of five hub targets (VEGFA, AKT1, EGFR, SRC, PIK3R1) to investigate the possible interactions between the compounds (quercetin, luteolin, kaempferide, kaempferol) and the active site of the targets. The binding affinity of each ligand-target was retrieved and luteolin and VEGFA were found to show the strongest binding affinity among the all binding results (Table 2). Besides, quercetin was found to interact with amino acids LEU66, CYS57, and LEU32 of VEGFA via a hydrogen bond (Figure 6(a)). In addition, luteolin was found to interact with amino acid PHE47 of VEGFA via *π*-bond and amino acids CYS60 and CYS26 via hydrogen bond (Figure 6(b)). More compound-amino acid residues interactions are presented in the supplementary materials (Table S2 and Figure S1).

### 3.2. Results of *In Vitro* Experiments

#### 3.2.1. Suppression of HCT116 Cell Growth *In Vitro*

**Figure 7:**
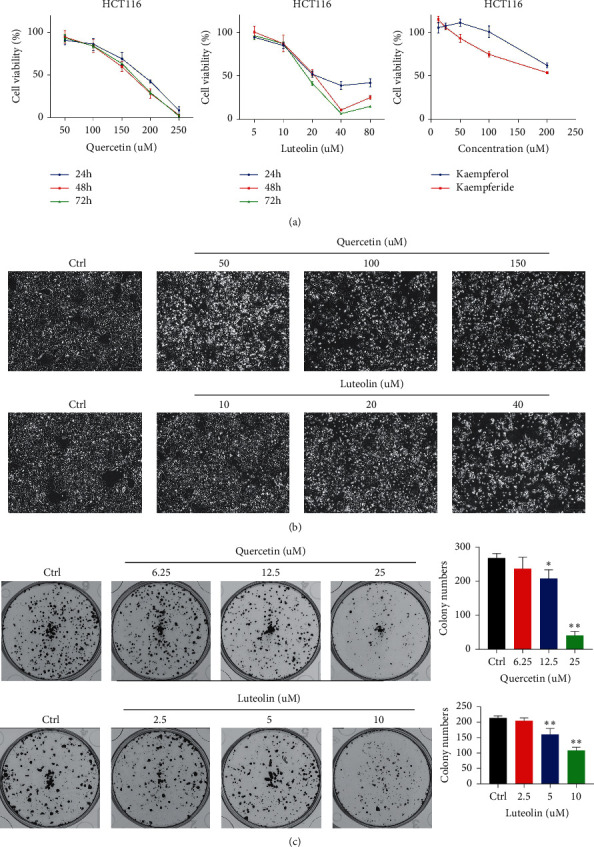
(a) Cell activity after intervention with compounds. (b) Cell morphology after treatment with compounds. (c) Number of cell clones after treatment with compounds (^*∗*^, *p* < 0.5; ^*∗∗*^, *p* < 0.01 vs. the ctrl group).

Quercetin and luteolin significantly inhibited cell proliferation of HCT116, whereas kaempferol and kaempferide had a lesser effect. After intervention with quercetin and luteolin for 24, 48, and 72 h, the IC_50_ of quercetin and luteolin was 168.3, 153.1, 160.7 *μ*M and 30.8, 20.6, 18.2 *μ*M, respectively (Figure 7(a)). According to Figure 7(b), the cells shrank and turned round with the increase of quercetin and luteolin concentration. Besides, the number of colonies formed by HCT116 was also reduced after the quercetin and luteolin were added. The trend of reduction was dose-dependent, as shown in Figure 7(c).

#### 3.2.2. The Potential Mechanisms of Anti-CC

**Figure 8:**
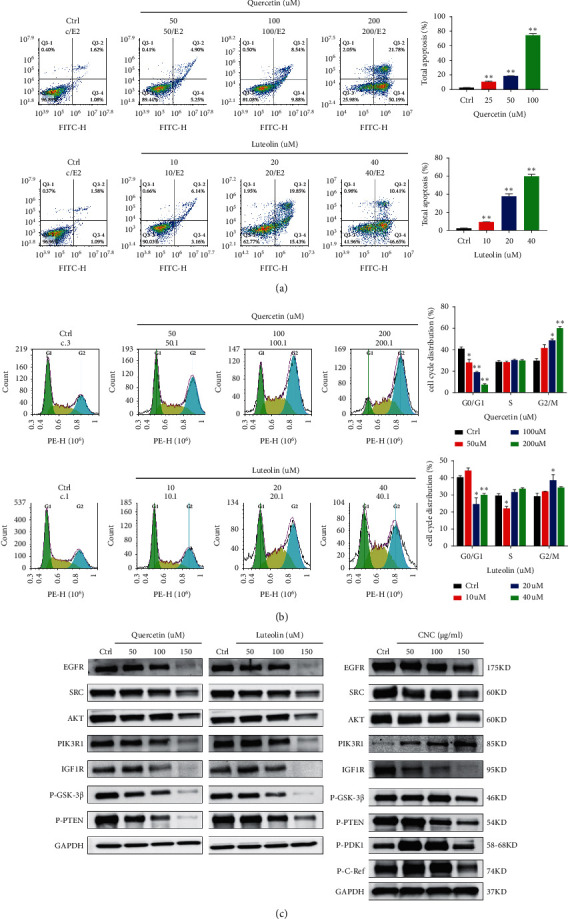
(a) The apoptosis assays of HCT116 incubated by quercetin and luteolin. (b) The cell cycle distribution of HCT116 was incubated with quercetin and luteolin. (c) The expression of related proteins in HCT116 was determined by western blotting (^*∗*^, *P* < 0.5; ^*∗∗*^, *P* < 0.01 vs. the ctrl group).

The effect of quercetin and luteolin on the apoptosis of HCT116 was determined based on the ability of Annexin V-FITC/PI to stain the phosphatidylserine on the outer membrane of cells and DNA fragmentation of the apoptotic cells [29, 30].  Figure 8(a) shows the increase of apoptotic cells with the increase of quercetin and luteolin concentration. The percentage of apoptotic cells in all treatment groups was higher than the control group. Moreover, quercetin and luteolin were also found to induce cell cycle arrest in HCT116. The result of propidium iodide (PI) staining shows that most of the HCT116 cells were arrested in G2/M phase after being treated with quercetin and luteolin (Figure 8(b)). Based on the results of CNC anti-CC network pharmacology, some core proteins and related proteins in the PI3K-Akt pathway were verified with *in vitro* experiments. HCT116 was intervened with quercetin and luteolin, or the extracts of CNC. After protein collection, the expression of proteins was measured by WB. All of them had downregulation effects on EGFR, SRC, AKT, IGF1R, P-GSK-3*β*, and P-PTEN. Interestingly, PIK3R1 was upregulated in the CNC group (Figure 8(c)). The results reveal that anti-CC effect of CNC was achieved by simultaneous multiple components and multiple targets.

## 4. Discussion

In recent years, the rise and development of TCM has been rapid. In clinical application, the advantage of the multicomponent, multipathway, and multitarget synergy of TCM has gradually become prominent over the single-target chemical drugs with high toxicity, side effects, and poor efficacy [31]. However, these advantages have also the key obstacle of TCM modernization because the mechanisms are difficult to identify. Nonetheless, the emergence of network pharmacology has provided new research ideas and technical means in TCM research to elucidate its mechanisms.

CC is the second deadliest malignant tumor globally, causing at least 600,000 deaths each year [32]. In this study, network pharmacology was carried out to elucidate the anti-CC mechanism of CNC by exploring the potential targets of CNC against CC. There were a total of 185 targets corresponding to the eight active components of CNC and a total of 1,366 disease-related targets identified. A total of 84 potential targets were obtained by taking the intersection. The majority of the compounds were flavonoids, such as quercetin, luteolin, kaempferide, and kaempferol, which have long been reported to have significant antitumor activity [33–36]. In this research, only eight compounds were found to meet the requirements of OB ≥30% and DL ≥ 0.18. Certainly, there were not only these eight compounds that contributed to the anticancer property of CNC. Other components, such as rutin, vitexin, and oleanolic acid which were reported to display anticancer activity, were also detected in CNC [37–39]. According to the PPI network, the top 10 proteins with the highest degree including VEGFA, AKT1, EGFR, SRC, and ESR1 were selected as the core targets. These proteins are well-known to be involved in multiple tumor and signal transduction pathways [40–42]. Besides, there were also correlations between the target proteins, which leads to the multiple targets and multiple pathways characteristics of CNC in suppressing CC.

AKT is a serine/threonine protein kinase, also known as protein kinase B. The AKT family mainly includes Akt1, Akt2, and Akt3, among which Akt1 plays a significant role in promoting tumor cell proliferation and inhibiting tumor cell metastasis [43]. VEGFA is a vascular endothelial growth factor (VEGF), which is the central regulator of neovascularization that leads to tumor development, metastasis, and recurrence. VEGF expression was detected in various cancers [44]. EGFR expression is associated with tumor cell proliferation, angiogenesis, tumor invasion, metastasis, and inhibition of apoptosis. Besides, its expression is also correlated with chemotherapy/radiation resistance, which results in poor prognosis [45]. Src family kinases represented by Src are a type of non-receptor protein tyrosine kinases. After the activation of c-Src protein which is expressed by the cell cancer genes, the mediated signal transduction pathways by MAPK, PI3K/Akt, and other pathways, will be overactivated and subsequently participate in the invasion and metastasis of a variety of malignant tumors [46]. In addition, the results of molecular docking show that the active components of CNC had a better affinity toward VEGFA and AKT1, which by lower binding energy results in a stronger binding ability. Enrichment analysis of the KEGG pathway unveils that multiple pathways were closely related to cancer, immune system, signal transduction, endocrine system, cell growth, and cell death. According to the “compound-target-pathway” network, the top five related disease pathways were cancer pathway, PI3K-Akt signaling pathway, proteoglycan in cancer, breast cancer, and Rap1 signaling pathway. Therefore, it can be predicted that CNC has a significant therapeutic effect on cancer. Thus, the PI3K-Akt signaling pathway was chosen to conduct a series of experiments for verification.

The hallmarks of cancer comprise six biological abilities acquired during the development of human tumors [47]. TCM with multitarget treatment mechanisms is believed to be a great potential drug for future cancer treatment in humans. Previous studies proved that CNC exhibited antitumor effects by inhibiting cell proliferation, blocking cell cycle, inducing cell apoptosis, etc. Based on the prediction in network pharmacology, an *in vitro* HCT116 cell model was established and the anti-CC effects of quercetin, luteolin, kaempferide, and kaempferol compounds were verified. The results show that quercetin and luteolin had a prominent inhibitory effect on the proliferation of HCT116, whereas kaempferide and kaempferol had no obvious inhibitory effect. After 48 h of drug treatment, the cell morphology, such as size, shape, and brightness, changed significantly. In addition, the cell clone formation was reduced gradually with the increase of concentration when the drug was applied to HCT116 for seven consecutive days. Results of Annexin V/PI double staining show that apoptosis rate was increased with the increasing dose, particularly at the early-stage apoptosis. Cell cycle experiments proved that quercetin could block the G2/M phase of the cells in a dose-dependent manner. In contrast, the inhibitory effect of luteolin in G2/M phase of cells was dose-dependent. In addition, the results of WB on some core protein pathways such as EGFR, SRC, AKT, IGF1R, and PI3K-Akt suggest that quercetin, luteolin, and CNC extracts had downregulated the protein expression of EGFR, SRC, AKT, IGF1R, P-GSK-3*β*, and P-PTEN. This finding was in accordance with many published studies [48–51]. It should be noted that simultaneous downregulation of PIK3R1 by quercetin and luteolin and upregulation of PIK3R1 by CNC extract may result in multiple actions of CNC crude.

The present study preliminarily elucidated the chemical components and mechanisms of the anti-CC effect of CNC. Compared with other anti-CC TCM, CNC has the characteristics of high efficiency and low toxicity. It has been applied as tea in folk to treat various diseases and has a long history. Modern pharmacological experiments also show that CNC has a wide range of safety and has no obvious toxic and side effects on mice. This research provides evidence to support the clinical use of CNC for the treatment of CC. Meanwhile, it is the first time systematically studying the mechanisms of CNC against CC using network pharmacology combined with an *in vitro* research method. It provides a scientific basis for elucidating the anticancer mechanisms of CNC. Due to the different cultural environments, the cells cultured *in vitro* cannot be entirely equal to those grown *in vivo*, and their biological behaviors are also different. Therefore, single cell culture is not enough to draw a general conclusion but should be combined with *in vivo* experiments to draw a more reliable conclusion.

## 5. Conclusions

In this study, an approach to combine network pharmacology and experimental verification was used to predict and verify the anti-CC mechanisms of CNC. Results of network pharmacology analysis show that quercetin, luteolin, kaempferide, and kaempferol were the main active constituents that suppress the activity of CC by regulating the VEGFA, AKT1, EGFR, and SRC proteins. GO and KEGG analysis found that PI3K-Akt and kinase signaling pathways were the main downstream mechanism pathways of the key targets. In addition, the results of molecular docking show that luteolin had the best binding ability with VEGFA, followed by quercetin. Furthermore, *in vitro* cell experiments proved that luteolin exhibited a stronger anti-CC effect than quercetin by inhibiting cell proliferation and colony formation, and inducing cell apoptosis and cell cycle arrest. The mechanism of anti-CC of luteolin may be related to its ability to inhibit the downstream signal transmission by targeting on the VEGFA and inhibiting the PI3K-Akt signaling pathway. The present research proved that CNC which is multicomponent, multitarget and multipathway could be effectively used to treat CC.

However, there are still some shortcomings in this study. The current work lacks *in vivo* experiments, which will be further verified in the follow-up research. In addition, the total extract of CNC should also be studied, rather than a few single chemical constituents.

## Figures and Tables

**Table 1:** The active ingredients information of CNC.

Compound name	PubChem CID	Molecular formula	Chemical structural
Quercetin	5280343	C_15_H_10_O_7_	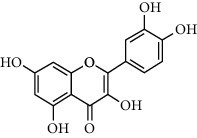
Kaempferol	5280863	C_15_H_10_O_6_	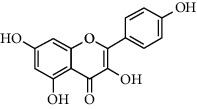
(+)-Catechin	9064	C_15_H_14_O_6_	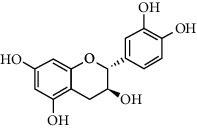
Luteolin	5280445	C_15_H_10_O_6_	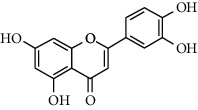
Kaempferide	5281666	C_16_H_12_O_6_	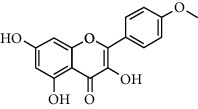
Beta-sitosterol	222284	C_29_H_50_O	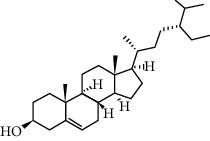
Alpha-L-Rhamnose	439710	C_6_H_12_O_5_	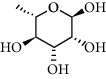
(E)-*α*-damascone	5366077	C_13_H_20_O	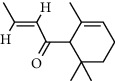

**Table 2:** Docking results of VEGFA with the active compounds.

Target	Compound	Binding energy/(kcal∙mol^−1^)
VEGFA	Quercetin	−6.18
VEGFA	Luteolin	−6.32
VEGFA	Kaempferide	−5.1
VEGFA	Kaempferol	−5.71

## Data Availability

The data used to support the findings of this study are available from Supplementary Materials.

## Abbreviations

TCM: Traditional Chinese medicine
CC: Colon cancer
CNC: *Camellia nitidissima* Chi
WB: Western blot
OB: Oral bioavailability
DL: Drug-likeness
TCMSP: Traditional Chinese Medicine Systems Pharmacology Database and Analysis Platform
PPI: Protein-protein interaction
MF: Molecular function
CCP: Cellular component
BP: Biology process.

## Supplementary Materials

Supplementary Materials Table S1: enrichment analysis of CNC‐CC targets based on Gene Ontology (GO) annotation. Table S2: docking results of VEGFA with the active compounds. Figure S1: docking results of VEGFA with the active compounds.
