# Supplementary material for: Identifying Active Compounds and Mechanism of Camellia nitidissima Chi on Anti-Colon Cancer by Network Pharmacology and Experimental Validation
Source: Evid Based Complement Alternat Med. 2021 Aug 26;2021:7169211. doi: 10.1155/2021/7169211 (PMC8413042; doi:10.1155/2021/7169211)
Supplement: Supplementary Materials — Table S1: enrichment analysis of CNC‐CC targets based on Gene Ontology (GO) annotation. Table S2: docking results of VEGFA with the active compounds. Figure S1: docking results of VEGFA with the active compounds. [file 7169211.f1.doc]

**Table S1.** Enrichment analysis of CNC‐CC targets based on Gene Ontology (GO) annotation.

| Ontology | ID | Term | p value | p.adjust | Count | Gene IDs |
| --- | --- | --- | --- | --- | --- | --- |
| BP | GO:0032870 | cellular response to hormone stimulus | -19.146 | -14.945 | 26 | PARP1|AKT1|APEX1|AR|CTSB|CTSL|EGFR|ESR1|ESR2|FLT3|GSK3B|IGF1R|INSR|KIT|NR3C2|PGR|PIK3R1|PPARD|PPARG|PTGER2|PTK2|PTPN1|RXRA|SRC|NR1H2|VDR |
| BP | GO:0071407 | cellular response to organic cyclic compound | -17.017 | -13.1184 | 22 | PARP1|AHR|AKT1|APEX1|AR|CFTR|CYP1B1|EGFR|ESR1|ESR2|FLT3|NR3C2|PGR|PIK3CG|PPARD|PPARG|PTGS2|RXRA|SLC6A4|SRC|NR1H2|VDR |
| BP | GO:0014065 | phosphatidylinositol 3-kinase signaling | -15.744 | -12.021 | 14 | AKT1|CAT|EGFR|F2|FLT3|IGF1R|INSR|KDR|KIT|PIK3CG|PIK3R1|PPARD|PTK2|SRC |
| BP | GO:0014068 | positive regulation of phosphatidylinositol 3-kinase signaling | -15.579 | -11.981 | 12 | CAT|F2|FLT3|IGF1R|INSR|KDR|KIT|PIK3CG|PIK3R1|PPARD|PTK2|SRC |
| BP | GO:0034599 | cellular response to oxidative stress | -15.301 | -11.8004 | 17 | PARP1|AKT1|APEX1|CAT|CDK1|CDK2|CYP1B1|DAPK1|EGFR|G6PD|MCL1|MET|MMP2|MMP3|MMP9|MPO|SRC |
| BP | GO:0014066 | regulation of phosphatidylinositol 3-kinase signaling | -15.198 | -11.777 | 13 | CAT|EGFR|F2|FLT3|IGF1R|INSR|KDR|KIT|PIK3CG|PIK3R1|PPARD|PTK2|SRC |
| BP | GO:0006979 | response to oxidative stress | -14.855 | -11.563 | 19 | PARP1|AKT1|APEX1|CAT|CDK1|CDK2|CYP1B1|DAPK1|EGFR|G6PD|MCL1|MET|MMP2|MMP3|MMP9|MPO|PTGS1|PTGS2|SRC |
| BP | GO:0043491 | protein kinase B signaling | -14.834 | -11.563 | 16 | AKT1|EGFR|ESR1|FGF1|FGF2|HSP90AA1|IGF1R|INSR|KDR|KIT|MET|PIK3CG|PIK3R1|PTK2|SRC|HPSE |
| BP | GO:0051897 | positive regulation of protein kinase B signaling | -14.809 | -11.5633 | 14 | EGFR|ESR1|FGF1|FGF2|HSP90AA1|IGF1R|INSR|KIT|MET|PIK3CG|PIK3R1|PTK2|SRC|HPSE |
| BP | GO:0071396 | cellular response to lipid | -14.745 | -11.545 | 21 | PARP1|AHR|AKT1|ALOX12|AR|CFTR|EGFR|ESR1|ESR2|FLT3|NR3C2|NOS2|PGR|PPARD|PPARG|PTGER2|RXRA|SLC6A4|SRC|NR1H2|VDR |
| CCP | GO:0031012 | extracellular matrix | -9.344 | -6.057 | 15 | CTSB|CTSD|CTSL|F2|FGF1|LGALS3|LGALS4|MMP2|MMP3|MMP9|MMP12|MMP13|PLG|VEGFA|HPSE |
| CCP | GO:0000323 | lytic vacuole | -6.884 | -4.074 | 14 | CAT|CFTR|CTSB|CTSD|CTSL|HSP90AA1|INSR|KIT|MPO|PIK3CG|SRC|TTR|TYR|HPSE |
| CCP | GO:0005764 | lysosome | -6.884 | -4.074 | 14 | CAT|CFTR|CTSB|CTSD|CTSL|HSP90AA1|INSR|KIT|MPO|PIK3CG|SRC|TTR|TYR|HPSE |
| CCP | GO:0043235 | receptor complex | -6.608 | -3.956 | 12 | AHR|ALK|EGFR|FLT3|IGF1R|INSR|KDR|KIT|MET|NR3C2|RXRA|VDR |
| CCP | GO:0031983 | vesicle lumen | -6.544 | -3.956 | 10 | ALOX5|CAT|CTSD|EGFR|HSP90AA1|MPO|PLG|TTR|VEGFA|HPSE |
| CCP | GO:0005773 | vacuole | -6.213 | -3.704 | 14 | CAT|CFTR|CTSB|CTSD|CTSL|HSP90AA1|INSR|KIT|MPO|PIK3CG|SRC|TTR|TYR|HPSE |
| CCP | GO:0098552 | side of membrane | -5.968 | -3.526 | 12 | ALOX15|CTSB|F2|G6PD|CXCR1|INSR|KIT|ABCB1|PLG|PTPN1|SRC|ABCG2 |
| CCP | GO:0034774 | secretory granule lumen | -5.751 | -3.395 | 9 | ALOX5|CAT|CTSD|HSP90AA1|MPO|PLG|TTR|VEGFA|HPSE |
| CCP | GO:0045121 | membrane raft | -5.685 | -3.395 | 9 | CTSD|EGFR|INSR|KDR|PTGS2|SLC6A4|SRC|ABCG2|HPSE |
| CCP | GO:0098857 | membrane microdomain | -5.674 | -3.395 | 9 | CTSD|EGFR|INSR|KDR|PTGS2|SLC6A4|SRC|ABCG2|HPSE |
| MF | GO:0004879 | nuclear receptor activity | -15.159 | -11.795 | 10 | AHR|AR|ESR1|ESR2|PGR|PPARD|PPARG|RXRA|NR1H2|VDR |
| MF | GO:0098531 | transcription factor activity, direct ligand regulated sequence-specific DNA binding | -15.159 | -11.795 | 10 | AHR|AR|ESR1|ESR2|PGR|PPARD|PPARG|RXRA|NR1H2|VDR |
| MF | GO:0004672 | protein kinase activity | -14.885 | -11.698 | 21 | AKT1|ALK|CDK1|CDK2|CDK6|DAPK1|EGFR|FLT3|GSK3B|IGF1R|INSR|KDR|KIT|MET|MYLK|PIK3CG|PLK1|PTK2|SRC|TOP1|AURKB |
| MF | GO:0003707 | steroid hormone receptor activity | -14.333 | -11.270 | 10 | AR|ESR1|ESR2|NR3C2|PGR|PPARD|PPARG|RXRA|NR1H2|VDR |
| MF | GO:0016773 | phosphotransferase activity, alcohol group as acceptor | -13.522 | -10.556 | 21 | AKT1|ALK|CDK1|CDK2|CDK6|DAPK1|EGFR|FLT3|GSK3B|IGF1R|INSR|KDR|KIT|MET|MYLK|PIK3CG|PLK1|PTK2|SRC|TOP1|AURKB |
| MF | GO:0016301 | kinase activity | -12.785 | -9.898 | 21 | AKT1|ALK|CDK1|CDK2|CDK6|DAPK1|EGFR|FLT3|GSK3B|IGF1R|INSR|KDR|KIT|MET|MYLK|PIK3CG|PLK1|PTK2|SRC|TOP1|AURKB |
| MF | GO:0004713 | protein tyrosine kinase activity | -10.259 | -7.479 | 10 | ALK|EGFR|FLT3|IGF1R|INSR|KDR|KIT|MET|PTK2|SRC |
| MF | GO:0004714 | transmembrane receptor protein tyrosine kinase activity | -10.241 | -7.479 | 8 | ALK|EGFR|FLT3|IGF1R|INSR|KDR|KIT|MET |
| MF | GO:0042562 | hormone binding | -9.960 | -7.249 | 9 | AR|EGFR|HSD17B1|IGF1R|INSR|PIK3R1|SHBG|TTR|VDR |
| MF | GO:0019199 | transmembrane receptor protein kinase activity | -9.400 | -6.735 | 8 | ALK|EGFR|FLT3|IGF1R|INSR|KDR|KIT|MET |

**Table S2.** Docking results of VEGFA with the active compounds.

| Target | Compound | Binding energy/ (kcal∙mol-1) | |
| --- | --- | --- | --- |
| AKT1 | Quercetin | -4.78 | |
| AKT1 | Luteolin | -4.23 | |
| AKT1 | Kaempferide | -4.8 | |
| AKT1 | Kaempferol | -4.14 | |
| EGFR | Quercetin | -4.13 | |
| EGFR | Luteolin | -3.94 | |
| EGFR | Kaempferide | -4.18 | |
| EGFR | Kaempferol | -3.65 | |
| SRC | Quercetin | -3.92 | |
| SRC | Luteolin | -4.07 | |
| SRC | Kaempferide | -4.42 | |
| SRC | Kaempferol | -4.87 | |
| PIK3R1 | Quercetin | -3.92 | |
| PIK3R1 | Luteolin | -3.82 | |
| PIK3R1 | Kaempferide | -3.06 | |
| PIK3R1 | Kaempferol | -3.16 |  |

**FigureS1.** Docking results of VEGFA with the active compounds.

| 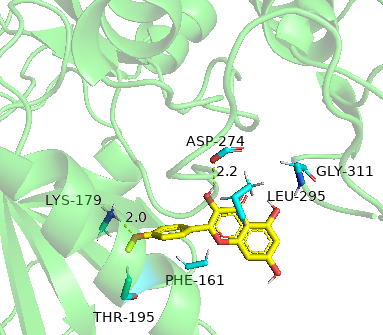 | 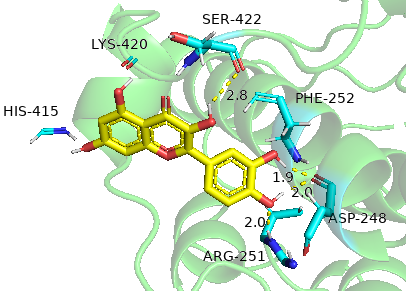 |
| --- | --- |
| A AKT1 (PDB ID: 4EKL) | |
| 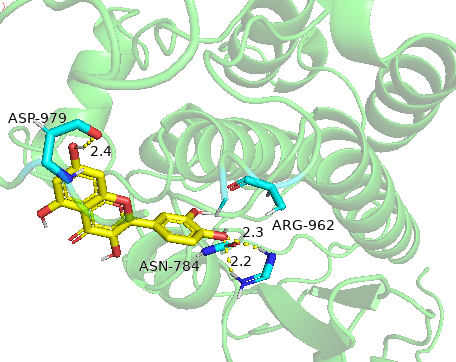 | 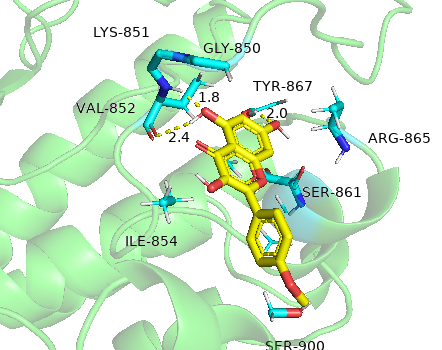 |
| B EGFR (PDB ID: 1M17) | |
| 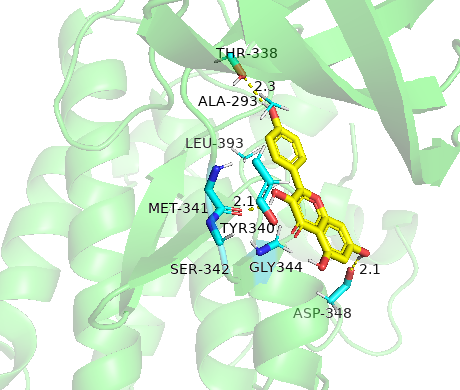 | 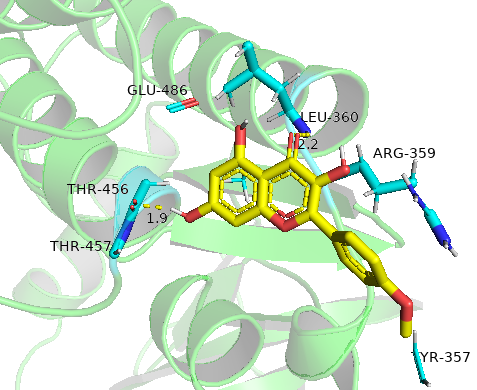 |
| C SRC (PDB ID: 2BDF) | |
| 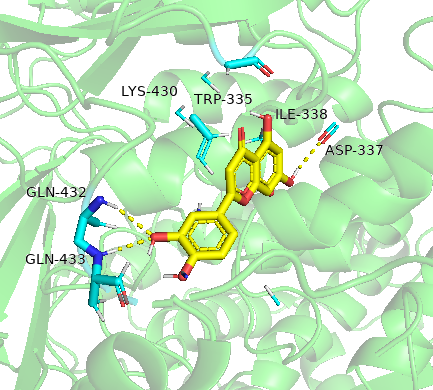 | 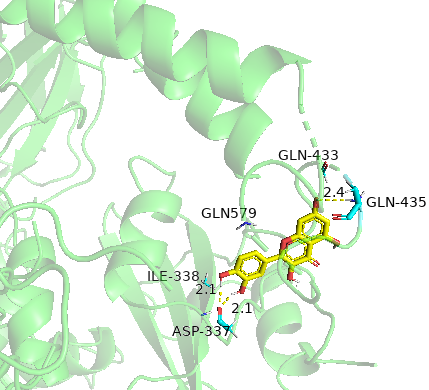 |
| D PIK3R1 (PDB ID: 3HHM) | |

(A represented the combination of AKT1 with kaempferid and quercetin; B represented the combination of EGFR with quercetin and kaempferid; C represented the combination of SRC with kaempferol and kaempferid; D represented the combination of PIK3R1 with luteolin and quercetin; )
